# Temperature-Dependent Clonal and Species-Level Growth Variation in *Spirodela*, *Landoltia*, *Lemna*, and Interspecific *Lemna* Hybrids

**DOI:** 10.3390/plants15111649

**Published:** 2026-05-27

**Authors:** Iride Mascheretti, Alessandra Mallardi, Claudia Liberatore, Tommaso Martinelli, Massimiliano Lauria

**Affiliations:** 1 Institute of Agricultural Biology and Biotechnology, Italian National Research Council (IBBA—CNR), Via A. Corti 12, 20133 Milano, Italy; iride.mascheretti@cnr.it (I.M.); alessandra.mallardi@ibba.cnr.it (A.M.); claudiamaria.liberatore@crea.gov.it (C.L.); 2 Consiglio per la Ricerca in Agricoltura e l’Analisi dell’Economia Agraria (CREA), Centro di Ricerca Difesa e Certificazione, Via di Lanciola 12, 50125 Firenze, Italy; tommaso.martinelli@crea.gov.it

**Keywords:** duckweed, temperature response, relative growth rate (RGR), clonal variation, intraspecific variation

## Abstract

Duckweeds are minute, fast-growing monocot aquatic plants that propagate clonally and combine high biomass productivity with a valuable biochemical composition (high-quality proteins, a favorable polyunsaturated fatty acid profile, and starch-rich tissues) and efficient nutrient uptake, making them attractive for feed/food, bioenergy, and wastewater-based phyto-bioremediation. Temperature is a key factor shaping duckweed growth, and selecting clones that perform well within specific thermal ranges can improve cultivation across different applications. Here, we screened 97 clones from the genera *Spirodela*, *Landoltia*, and *Lemna*, including the hybrids *Lemna* × *japonica* and *Lemna* × *mediterranea*, under warm (WC; 30/25 °C) and relative cool (CC; 20/16 °C) conditions. Relative growth rate (RGR) ranged from 0.150 to 0.338 day^−1^ under WC and from 0.113 to 0.318 day^−1^ under CC, revealing strong interspecific and intraspecific variation. While WC generally promoted higher growth than CC, notable exceptions occurred at both interspecific and intraspecific levels. Tests under more extreme regimes (EWC; 35/30 °C; ECC; 16/12 °C) confirmed strong clone-specific responses, with some clones maintaining or improving growth under EWC relative to WC, whereas ECC generally reduced growth relative to CC. Climatic provenance was a weak predictor of performance, showing limited correspondence between RGR and mean annual temperature at the site of origin. Overall, these results highlight the value of within-species phenotyping across relevant temperature regimes to identify high-performing duckweed material for applied use.

## 1. Introduction

The term duckweed refers to a group of monocotyledonous, free-floating aquatic plants (*Lemnaceae*), comprising five genera (*Spirodela*, *Landoltia*, *Lemna*, *Wolffia*, and *Wolffiella*) and 35 species [1]. However, duckweed taxonomy is complex and still under revision in light of recently identified interspecific hybrids and cryptic species [2,3].

A key feature of this family is its extremely rapid clonal propagation via vegetative budding, which enables remarkable biomass yields. This exceptional growth, together with their minute size, has fueled increasing interest in duckweeds for several applications, including phyto-bioremediation, bioenergy production, and the production of feed and food. Furthermore, duckweeds have emerged as an attractive model system for biotechnological purposes [4,5]. In recent years, duckweeds have also become a major focus of omics-based research, leading to high-quality genome assemblies for species of different genera. These genomic and transcriptomic resources have accelerated studies on duckweed biology, physiology, and evolutionary dynamics, providing insights into the genetic basis of its rapid growth, reduced morphology, and stress responses [6,7,8,9,10,11].

The high nutritional value of duckweed is largely driven by its high-quality protein content, including indispensable amino acids for human nutrition [4,5,12,13,14]. Duckweed also shows a favorable polyunsaturated fatty acid profile, comparable to that of legume flours, making it an attractive resource for feed and food production [4,12,13,14,15]. In addition, duckweed can provide starch-rich biomass for downstream applications such as bioethanol production. This process is facilitated by the absence of lignin and the low cellulose content, which improve biomass conversion efficiency [16,17,18]. Importantly, starch accumulation can be enhanced under specific growth conditions, including salt stress [19], nutrient starvation [20], and high light intensity [21]. Notably, a recent study on *Landoltia punctata* showed that combining nutrient starvation with uniconazole (a gibberellin biosynthesis inhibitor) and elevated CO_2_ levels increased starch content to up to 76% of dry weight [18].

Depending on the end use, duckweeds can be cultivated in both outdoor and indoor systems. Outdoor systems, such as ponds and lagoons, are widely adopted because they are inexpensive, operationally simple, and easily scalable; however, they require large land areas and are strongly affected by environmental fluctuations, which can limit year-round cultivation in some regions [5,22]. In contrast, indoor cultivation systems include high-tech configurations such as controlled lagoon-based systems in polytunnels and stacked flow-through vertical systems, which require little horizontal space and are therefore well suited to urban environments [5,22]. These systems enable precise control of key cultivation parameters (e.g., temperature, light, air circulation and sterility), making them especially suitable for pharmaceutical applications and for producing biomass intended for human consumption [22,23]. Moreover, indoor systems support year-round cultivation independent of seasonal constraints and can accommodate species with narrow environmental requirements, such as *Wolffia* spp., which are naturally restricted to tropical regions and are widely consumed in Asian diets [12,13].

Duckweed growth and physiology are influenced by multiple environmental and nutritional factors, including light regime, temperature, nutrient availability (nitrogen and phosphorus), water chemistry (pH and salinity), CO_2_ concentration, and exposure to pollutants or heavy metals [24,25,26,27,28,29,30]. Among these, temperature is particularly important because it modulates photosynthesis, nutrient uptake, cell division, and overall metabolism, thereby directly affecting biomass accumulation and productivity [24,31,32]. A recent meta-analysis reported an optimal growth range between 20 °C and 30 °C, although species differ in their performance patterns [33]. For example, *Lemna* spp. tolerate a broader temperature range (11.4–38.1 °C) than *La*. *punctata* (18.1–32.3 °C) and *Spirodela* spp. (19.0–29.2 °C). Outside these ranges, growth may still occur, but relative growth rate (RGR) typically declines and survival can be compromised under extreme temperatures [32,33,34,35].

Regardless of the final end use, optimizing duckweed-based production systems requires not only fine-tuning cultivation parameters, but also selection of suitable species and clones. Temperature is therefore a key selective factor, as clones can differ markedly in growth performance and thermal tolerance even under controlled conditions [33]. Here, we conducted a screening of 97 duckweed clones from different species under varying temperature conditions. The clones belong to the *Spirodela*, *Landoltia*, and *Lemna* genera, and include the two recently identified interspecific hybrids *Lemna* × *japonica* and *Lemna* × *mediterranea* [36,37]. This screening provides a comparative framework to identify temperature-resilient, high-performing clones for optimized cultivation strategies.

## 2. Results

### 2.1. Clone Selection

**Figure 1:**
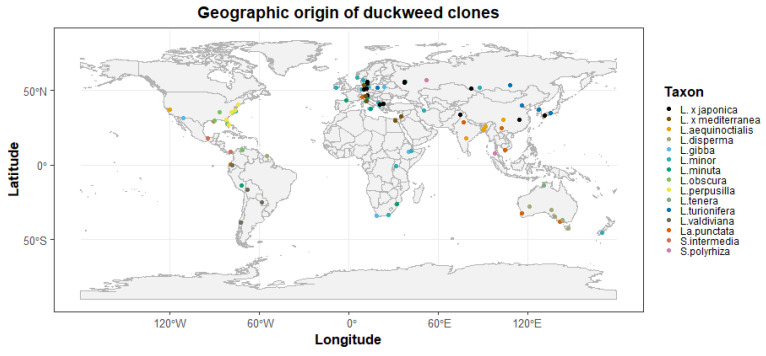
Geographic origin of the 97 duckweed clones analysed. Genus abbreviations: *Spirodela* (S.), *Landoltia* (La.), and *Lemna* (L.). Interspecific hybrids are indicated by the symbol ×.

Duckweeds can grow and survive within a temperature range of 5 °C to 40 °C, although the optimal range for various species and clones has been evaluated to be between 20 °C and 30 °C [32,33,34,38]. Based on that, we selected 97 clones originated from different world areas characterized by boreal, temperate, subtropical or tropical climate with the aim to test their growth performance under different controlled temperature conditions (Figure 1, Table S1). Overall, the selected clones are representative of 13 species belonging to three genera (*Spirodela*, *Landoltia* and *Lemna*), and two recently identified hybrids, *L.* × *japonica* and *L.* × *mediterranea* [36,37]. These hybrids allowed us to explore for potential heterosis (hybrid vigor), i.e., higher RGR and/or reduced sensitivity to contrasting temperature regimes relative to the parental species. Clones originated primarily from Europe (35), followed by Asia (20), North America (14), Oceania and South America (10 clones each) and Africa (eight). The northernmost and the southernmost clones were respectively from Norway (9495) and New Zealand (7766); both belonged to *Lemna minor* (Figure 1, Table S1). The most representative group belonged to the *Lemna* genus (60/97 clones), followed by *Spirodela* (10/97 clones) and *Landoltia* (8/97 clones). Fourteen and five clones belonged to the hybrid *L.* × *japonica* and *L.* × *mediterranea*, respectively (Figure 1, Table S1).

### 2.2. Growth Performance of Duckweed Under Near-Optimal and Optimal Temperature Conditions

To assess duckweed growth performance, we initially applied two temperature regimes overlapping near-optimal and optimal conditions: warm conditions (WC; 30/25 °C) and relatively cool conditions (CC; 20/16 °C). Growth performance of each clone was expressed as relative growth rate (RGR), calculated from dry weight (DW) measurements.

**Figure 2:**
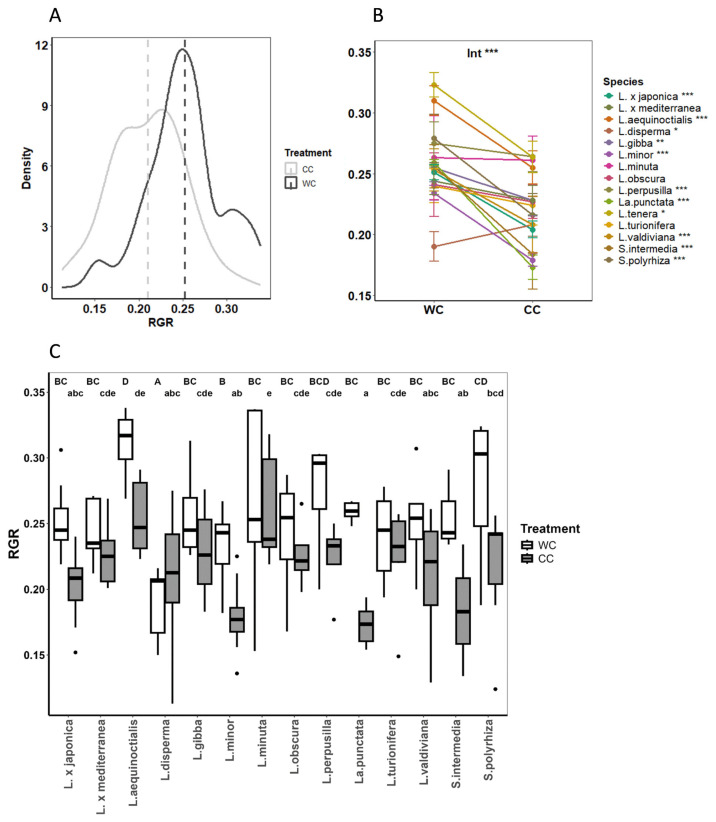
(**A**) Density distributions of RGR values of all clones analyzed under warm and cool conditions (WC and CC). Dashed lines indicate the mean of each distribution. (**B**) Mean RGR (±SE) of each species and hybrid under WC and CC (species-level means across clones). Asterisks next to species names indicate statistically significant differences between WC and CC for that species (* *p* < 0.05, ** *p* < 0.01, *** *p* < 0.001). “Int.” indicates a significant species × treatment interaction (two-way ANOVA). (**C**) Boxplot showing intra-species variation in RGR under WC and CC treatment. Letters indicate statistical significance according to the ANOVA test: boxes labeled with different letters differ significantly, whereas boxes sharing the same letter do not differ significantly. Uppercase and lowercase letters refer to WC and CC, respectively.

**Figure 3:**
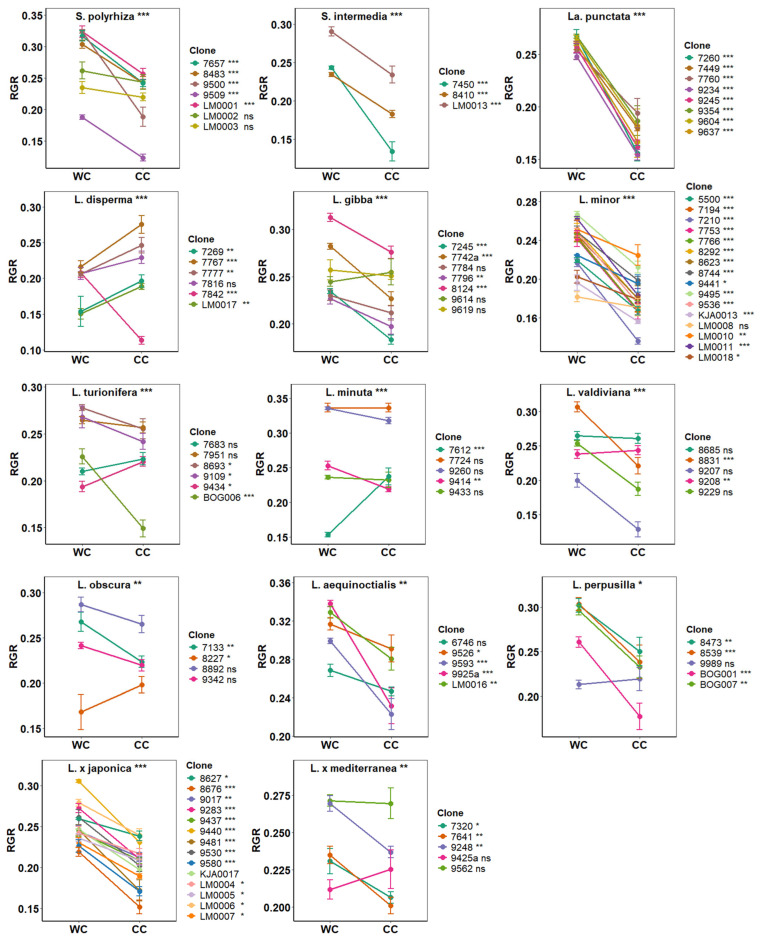
Mean RGR (±SE) of individual clones within each species/hybrid under WC and CC. Asterisks next to species names indicate a statistically significant clone × treatment interaction within that species/hybrid (i.e., heterogeneous clone responses to temperature). Asterisks next to clone codes indicate statistically significant differences between WC and CC for that clone (* *p* < 0.05, ** *p* < 0.01, *** *p* < 0.001; ns, not significant). All statistical tests were performed within species/hybrids; this figure does not represent interspecific comparisons.

Overall, RGR values were generally higher under WC than under CC (Figure 2A), with an average of 0.252 day^−1^ and 0.210 day^−1^, respectively. Genus-stratified density plots (Figure S1) indicate that genera differ in both central tendency and spread (e.g., *Landoltia* shows a relatively narrow distribution under WC and a shift under CC), suggesting that the multimodality in Figure 2A partly reflects taxonomic heterogeneity. Individual clone RGR values ranged from 0.150 day^−1^ (LM0017, *Lemna disperma*) to 0.338 day^−1^ (9925a, *Lemna aequinoctialis*) under WC, and from 0.113 day^−1^ (7842, *L. disperma*) to 0.318 day^−1^ (9260, *Lemna minuta*) under CC (Table S2). A two-way ANOVA showed a significant species × treatment interaction (*p* < 0.001), indicating species-specific thermal responses (Figure 2B; species means ± SE across clones). Clone-level responses are presented in Figure 3.

Simple main effect analyses indicated that, with the exception of *L.* × *mediterranea*, *L. minuta*, *Lemna obscura*, and *Lemna turionifera*, all species showed statistically significant differences in RGR between the two temperature conditions (Figure 2B). Interestingly, in contrast to the general growth pattern, *L. disperma* performed better under CC than WC (Figure 2B).

Indeed, on average, *L. disperma* exhibited the lowest growth performance (0.190 days^−1^) under WC and differed statistically from all other species (Figure 2C, Table S2). Differently, *L. aequinoctialis* (0.310 day^−1^) exhibited the highest performance and differed statistically from most of the species/hybrids analyzed. *Lemna tenera*, despite a good performance (0.323 day^−1^), was excluded from the statistical analysis because only one clone was available in our collection at the time of the experiments (Table S2). Under CC, *La. punctata* (0.173 day^−1^) and *L. minuta* (0.261 day^−1^) showed the lowest and highest average RGR values, respectively (Figure 2C, Table S2).

Significant variation, however, was observed intra-species (Figure 2C and Figure 3 , Table S2). For instance, under WC, *L. minuta* included clones ranging from 0.337 day^−1^ (7724) to 0.153 day^−1^ (7612), *Spirodela polyrhiza* showed clones ranging from 0.324 day^−1^ (LM0001 and 9500) to 0.188 day^−1^ (9509), and *L. obscura* included clones ranging from 0.287 day^−1^ (8892) to 0.168 day^−1^ (8227). Likewise, under CC, the variation among *L. disperma* clones ranged from 0.275 day^−1^ (7767) to 0.113 day^−1^ (7842), *S. polyrizha* ranged from 0.256 day^−1^ (LM0001) to 0.124 day^−1^ (9509), and *Lemna valdiviana* ranged from 0.261 day^−1^ (8685) to 0.188 day^−1^ (9229).

With the exception of *La. punctata*, whose clones all respond similarly to temperature treatment, a significant clone × treatment interaction was observed (Figure 3). Notably, although each species or hybrid exhibited a consistent overall RGR pattern between WC and CC (Figure 2B), clear deviations emerged at the clone level, indicating that individual clones within the same species may respond differently to temperature changes. Perhaps the most striking example was clone 7842 (*L. disperma*), which, unlike the other five clones, performed better under WC than CC. Similarly, clone 7612 (*L. minuta*) also showed higher performance under CC, whereas the remaining clones were little-affected or unaffected by the two temperature regimes (Figure 3). This pattern of minimal response was also observed for other clones across different species (e.g., *L. valdiviana*, *Lemna gibba*, *Lemna perpusilla*, and *L.* × *mediterranea*), highlighting that some clones, under our experimental conditions, maintained relatively stable growth rates over a wide temperature range (16–30 °C). Of note, is the contrasting behavior observed between the two hybrids: although almost three times as many clones of *L.* × *japonica* were analyzed as of *L.* × *mediterranea*, all 14 clones of *L.* × *japonica* displayed a similar growth pattern, whereas the five clones of *L.* × *mediterranea* exhibited a more variable response (Figure 3). Moreover, the two hybrids *L.* × *japonica* (derived from *L. minor* and *L. turionifera*), and *L.* × *mediterranea* (derived from *L. minor* and *L. gibba*) were not consistently more performant than their parental species across treatments, providing no clear evidence of heterosis in terms of RGR under our experimental conditions.

### 2.3. Growth Performance Under Severe Temperature Conditions

Different clones were tested under more extreme temperature regimes, namely an extreme warm condition (EWC, 35/30 °C) and an extreme cool condition (ECC, 16/12 °C). In total, 36 and 20 clones were included in the EWC and ECC experiments, respectively. With few exceptions, the selection primarily focused on clones that performed best under WC and CC. The EWC treatment encompassed all 13 species and the two hybrids previously analyzed under WC, including one to three clones per species/hybrid. For the ECC experiment, 20 clones were chosen, representing at least one clone per species/hybrid (except for *S. intermedia*). Of the 36 clones analyzed, 19 were tested under both EWC and ECC.

Under EWC, the mean RGR of the clone subgroup was lower than under WC (0.249 vs. 0.277 day^−1^; Table S3). However, among the 36 clones analyzed, 11 (30%) showed comparable or higher RGR under EWC than under WC, with increases ranging from 1% to 21%. These clones belonged to five species/hybrids: *S. polyrhiza* (2/3), *L. aequinoctialis* (3/3), *L. perpusilla* (2/2), *L.* × *mediterranea* (2/3), *L.* × *japonica* (2/3; Table S3). *L. aequinoctialis* included the best performing clone (9925a) with an RGR of 0.384 day^−1^, whereas *L. perpusilla* (8473) showed the highest RGR delta between EWC and WC (21%). The remaining 23 clones were characterized by a reduction in RGR ranging from 4.7 to 53.4% for *L. turionifera* (8693) and *L. minuta* (7724), respectively. The poorest performances under EWC were recorded for *L. minuta* (7612, 0.091 day^−1^) and *L. disperma* (7816, 0.116 day^−1^), with only one out of the three *L. disperma* clones surviving under these conditions.

As observed under EWC, the mean RGR of the 20 clones analyzed under ECC was lower than that recorded under CC (0.202 vs. 0.242 day^−1^; Table S3). Only one clone, *L. perpusilla* (8473), exhibited an RGR comparable to that under CC (0.251 vs. 0.250 day^−1^). Notably, this clone also ranked among the best performers under EWC, with an even higher RGR (0.365 day^−1^) than under WC (0.302 day^−1^), confirming its capacity to sustain rapid growth across a wide temperature range (12–35 °C). The best- and worst-performing clones under ECC were *L. minuta* (7724; 0.263 day^−1^) and *L. tenera* (9024; 0.134 day^−1^), respectively, and the overall RGR reduction observed ranged from 10% (*L. minor*; LM0010) to 49% (*L. tenera*; 9024).

**Figure 4:**
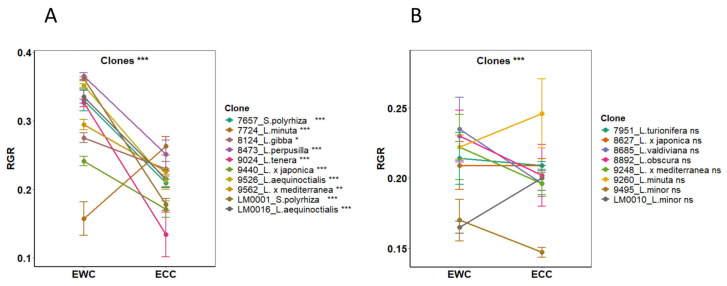
(**A**,**B**) Mean RGR (±SE) of specie and hybrid clones tested under both EWC and ECC. The two figures refer to a single statistical analysis and were split for clarity. *, **, and *** indicate *p* < 0.05, 0.01, and 0.001, respectively; ns indicates no significant difference. The asterisk beside graph title (clones) indicates the statistically significant clone × treatment interaction highlighting a different temperature effect on RGR in the single clones tested. The asterisks beside the clone code indicate statistically significant differences between EWC and ECC for that clone.

A significant clone × treatment interaction (*p* < 0.001) was observed: ten clones showed significant differences between EWC and ECC temperatures (Figure 4A), whereas the remaining clones did not (Figure 4B). Among the clones that showed statistically significant differences between EWC and ECC, high temperatures appeared more detrimental than low temperatures in only one case (*L. minuta*; 7724). Overall, these results indicate that the two temperature treatments exerted distinct effects on the selected clones.

### 2.4. Correlation Between Duckweed Origin and Temperature Response

To investigate whether RGR was associated with climatic conditions at the sites of origin of the clones, potentially consistent with adaptation to local thermal regimes, we retrieved 30-year (1970–2000) average temperature records. When all clones were analyzed together, no significant relationship was detected between the RGR measured under either WC or CC and the mean temperature of the site of origin (Figure S2A,B). RGR values were highly dispersed across the temperature gradient, indicating that temperature of the site of origin alone is a poor predictor of growth under our common garden conditions. Despite the absence of an overall trend, substantial variability was observed among clones, suggesting strong genotype-specific responses.

**Figure 5:**
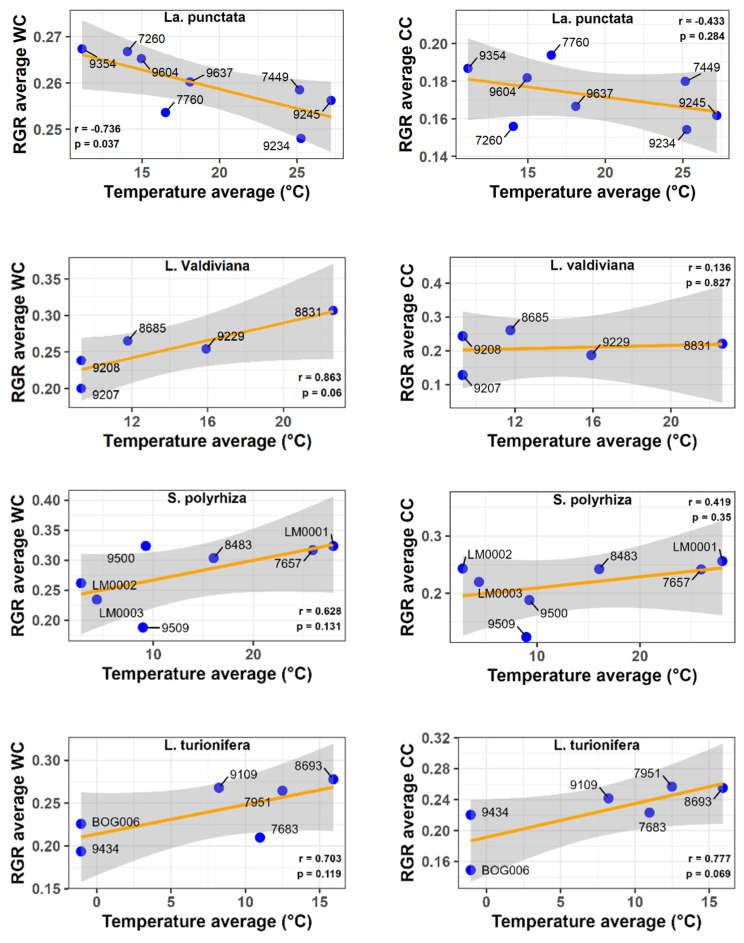
Representative examples illustrating species-specific relationships between mean relative growth rate (RGR) and the average temperature of the site of origin under WC (**left**) and CC (**right**) conditions. Blue circles represent individual clones, and the orange line indicates the linear regression. The grey shaded area represents the 95% confidence interval of the regression line. Pearson’s correlation coefficient (r) and associated *p*-values are shown.

At the species level, analyses suggested weak and species-specific patterns (Figure 5, Table S4). Across the species analyzed, only one statistically significant correlation was detected (*La. punctata* under WC), whereas other species showed non-significant or inconsistent trends across temperature treatments. In *La. punctata*, accessions originating from colder regions exhibited higher RGR under WC, resulting in a negative association between RGR and temperature of the site of origin. By contrast, although not statistically significant, *L. valdiviana*, *S. polyrhiza*, and *L. turionifera* tended to show positive associations between RGR and temperature of origin under WC (Figure 5).

## 3. Discussion

Temperature is a key determinant of duckweed growth, influencing major physiological processes and biomass production; however, temperature-dependent growth assessments based on extensive clonal panels remain limited and often rely on a small number of species or representative clones [30,32,34,35,39,40,41,42,43,44]. In this study, we investigated the growth performance of 97 duckweed clones, as determined by relative growth rate (RGR), from the genera *Spirodela*, *Landoltia*, and *Lemna* under near-optimal (CC), optimal (WC) and unfavorable temperature conditions (ECC and EWC). Although *Wolffia* has been demonstrated to be highly relevant for human consumption [4,13], it was not included in this study at this time because our dry-weight–based RGR assay was standardized using an inoculum defined by a fixed number of fronds, an approach that is technically less reliable for *Wolffia* due to its minute size (see Section 4). Because RGR was calculated using the same approach for all clones under identical experimental conditions, interspecific comparisons are interpreted here as standardized performance within our experimental framework rather than as general species-level conclusions. It is also important to note that RGR was calculated on a dry-weight (DW) basis and therefore reflects biomass accumulation rather than frond multiplication per se. Increased starch accumulation under non-optimal temperatures has been reported in several duckweed species [24,45,46], likely as part of a stress-related response [47]. Consequently, temperature-dependent changes in tissue composition could contribute to similar RGR values despite different rates of clonal propagation.

Ziegler et al. [48] examined the growth performance of 39 duckweed clones belonging to 13 species across the five recognized genera, including the three genera also investigated here (*Spirodela*, *Landoltia* and *Lemna*). Although all clones were grown at a constant 25 °C, the study highlighted pronounced inter- and intraspecific differences in growth performance. Based on these data, the authors concluded that genus- or species-level classification is not a reliable predictor of the growth potential of individual duckweed clones. A similar conclusion was reached by Sree et al. [49], who investigated 25 clones representing all 11 species of the genus *Wolffia*, also grown at a constant 25 °C. The same conclusion is supported by the findings of the present study, which is based on a substantially larger dataset and multiple temperature regimes. Although a quantitative comparison between our results and those of Ziegler et al. [48] is limited by differences in experimental design and partial overlap in clone identity, a limitation shared with other studies, both studies highlight pronounced interspecific variability, largely attributable to differences in clone-level performance.

Consistent with this interpretation, substantial intraspecific variation in RGR was observed, with best-to-worst clone ratios exceeding a value of two in some cases, such as in *S. polyrhiza*, *L. disperma* and *L. valdiviana* under CC, and *L. minuta* under WC. Overall, intraspecific differences in RGR were generally greater under CC than under WC, suggesting that low temperatures may accentuate interclonal differences in thermal tolerance and growth plasticity, thereby widening the performance gap between genotypes. This pattern is consistent with the idea that sub-optimal conditions increase the expression of genetic and physiological constraints (e.g., reduced metabolic flexibility or photosynthetic efficiency), leading to a stronger divergence among clones [46]. Notably, in line with the findings of Ziegler et al. [48], *La. punctata* was the only species that did not exhibit a statistically significant clone × treatment interaction, suggesting a comparatively uniform response of its clones to temperature treatments and reduced intraspecific variability relative to the other species analyzed. This comparatively uniform response in *La. punctata* may reflect relatively limited genetic diversity among the accessions included in our panel and/or a more canalized thermal response in this species, resulting in reduced genotype-by-temperature interactions. Consistent with this possibility, low within-population genetic diversity in *La. punctata* has been reported in some Asian datasets [50,51]. However, the absence of a significant clone × treatment interaction does not necessarily imply genetic homogeneity, as it may reflect the limited breadth of clonal sampling within this species. Analysis of a larger number of *La. punctata* accessions, coupled with genome-wide data, would be needed to determine whether this pattern is consistent and whether additional variation in thermal plasticity emerges.

Overall, as expected, WC tended to promote higher RGR than CC; however, notable exceptions were observed at both inter- and intraspecific levels. At the species level, *L. disperma* was the only species that, on average, performed better under CC. However, this growth pattern does not necessarily indicate a general preference for cooler over warmer conditions, as at least one clone (7842) exhibited an opposite behavior. Five of the six clones analyzed for this species originated from Oceania, including clone 7842. Given the long-term geographical isolation of this region, large-scale biogeographic separation alone is therefore unlikely to explain the overall tendency of *L. disperma* to perform better under CC. Similar contrasting responses among clones were detected in other species as well, including *L. turionifera* and *L. minuta*. Moreover, with the exception of *S. intermedia*, *La. punctata* and *L.* × *japonica*, all species/hybrids included several clones for which RGR differences between WC and CC were not statistically significant, indicating a capacity to maintain growth across a relatively wide range of temperatures (16–30 °C). While in some cases the lack of significant differences between WC and CC may reflect high variability among replicates, this explanation is unlikely to apply universally, as several clones showed comparable low standard error values across temperature treatments.

Together, these results underscore the importance of considering intraspecific variation when interpreting species-level growth responses across contrasting environmental conditions. Thus, although our study suggests that some species may, on average, perform better under a specific thermal regime, we cannot rule out the possibility that a more extensive species clonal screening could identify clones well suited to either temperature regime. Nevertheless, our screening pinpointed clones that performed well under both WC and CC, making them strong candidates for practical use across contrasting temperature conditions. Notably, among all clones analyzed, *L. minuta* (9260) was not only among the best-performing clones under both WC and CC, but also showed minimal differences (5%) between the two conditions.

The use of two recently identified hybrids, *L.* × *japonica* (*L. minor* × *L. turionifera*) and *L.* × *mediterranea* (*L. minor* × *L. gibba*; [36,37]), allowed us to test the effect of heterosis (hybrid vigor), which is typically defined as the superior performance of hybrids relative to their parental lines in terms of yield and, in some cases, enhanced tolerance to stresses [19]. In our case, none of the hybrids consistently performed better than their parental species across temperature treatments, nor were they generally superior to rest of the clones analyzed providing no clear evidence of heterosis in RGR under our experimental conditions.

The use of more extreme conditions (EWC and ECC) on a subset of selected clones revealed two main patterns. First, all clones except one, *L. perpusilla* (8473), performed worse under ECC than under CC, with percentage reductions ranging from 11% to 49%. Moreover, *L. disperma* confirmed its sensitivity to high temperature, as growth under EWC was essentially impaired. In contrast, under EWC, we found clones that exhibited comparable or higher RGR values than those observed under WC. Second, when RGR was compared between the two thermal treatments (EWC and ECC), statistically significant differences were detected only for a subset of the clones analyzed, suggesting that out of their optimal temperature range, both EWC and ECC negatively affected growth performance. Notably, *L. minuta* (7244) was the only clone that performed significantly better under ECC than under EWC. Overall, these results indicate that although extreme temperatures can negatively affect duckweed growth [33,34,41], the magnitude and even the direction of this effect depend strongly on clonal identity.

Considering the geographical origin of the material investigated in this study, variation in thermal responses might reflect eco-geographical differentiation, whereby clones may be associated with the climatic conditions at their site of origin. This expectation is in line with the concept of local adaptation, whereby populations tend to achieve higher fitness in their native environments than foreign populations [52]. However, meta-analyses across plant reciprocal-transplant studies show that although local adaptation is common, it is less frequent than is generally believed [53]. Consistent with this, in *S. polyrhiza* the timing and extent of turion production vary along latitudinal gradients, with northern populations showing earlier temperature-induced turion formation than southern ones [54]. Moreover, among-clone differences in turion formation have been associated with climatic variables at the site of origin, including mean annual temperature [54,55].

When considering all clones analyzed under our experimental conditions, we found limited evidence of a strong association between RGR under WC and CC and environmental conditions at the site of origin, as correlations were generally weak. Only in a few cases did the patterns suggest a potential correspondence. At the species level, only *La. punctata* showed a statistically significant correlation between RGR under WC and mean temperature at the site of origin. Unexpectedly, the relationship was negative, with higher RGR values in accessions originating from colder regions. In addition, moderate-to-strong positive correlations were observed for *L. valdiviana*, *L. turionifera*, and *S. polyrhiza*, although these did not reach statistical significance. Given the small sample sizes, these patterns should be interpreted cautiously, and a broader screening would be needed to clarify whether these trends are consistent.

Several factors may account for these generally weak associations. Relying on a single predictor, such as annual temperatures, may not capture the environmental complexity of the sites of origin, which can differ markedly in seasonality and microclimate. In addition to temperature, photoperiod, light intensity, and nutrient concentrations (e.g., N and P) are key determinants of duckweed growth responses [29,30,34,44,56,57]. Consistently, Kuehdorf et al. [55] reported that turion yield was only weakly associated with individual climatic variables at the site of origin, whereas models combining multiple climatic parameters (multiple regression) provided a better fit to turion yield. Moreover, standardized laboratory conditions may mask provenance-related differences, leading to large performance differences even among accessions from sites with similar mean annual temperatures. Indeed, clones from geographical locations with similar mean annual temperatures displayed more than twofold differences in RGR, e.g., *L. aequinoctialis* 9925a vs. *L. disperma* LM0017 under WC and *L. minuta* 9260 vs. *L. disperma* 7842 under CC. Our experimental conditions may also have emphasized broad thermal tolerance or phenotypic plasticity rather than site-specific adaptation, as some accessions from colder regions maintained a high RGR under warm conditions (e.g., *La. punctata*). This interpretation is further supported by within-species variation among accessions collected from the same geographical area or even the same locality; for example, *S. polyrhiza* 9500 vs. 9509 (same area), *L. valdiviana* 9207 vs. 9208 (same locality), and *L. turionifera* BOG006 vs. 9434 (same locality) showed differences in RGR highlighting high clonal diversity in the same locality. Finally, long-term maintenance under constant stock-collection conditions, together with the repeated sterilization cycles required for aseptic culture, may impose stress and recurrent propagation bottlenecks that promote stochastic clonal sorting, potentially leading to the enrichment or random fixation of somatic mutations and/or epigenetic variants that affect performance [58,59,60,61].

Because our study was conducted under common-garden laboratory conditions, the differences observed here are best interpreted as variation in thermal reaction norms among the analyzed clones (i.e., genotype-dependent responses across environments) rather than as direct evidence of local adaptation in natural environments [62]. Local adaptation generally requires field-realistic tests (e.g., reciprocal transplant approaches), and its frequency/strength varies widely across plant studies [52,53]. Accordingly, translating clone-specific thermal performance to natural settings will likely require experiments that better approximate fluctuating temperatures and interacting cues (e.g., photoperiod and nutrients), beyond provenance-based expectations alone.

Taken together, the selection of high-performing material for applied purposes may benefit more from direct phenotyping across relevant temperature regimes than from provenance-based expectations alone. At the same time, detecting eco-geographical structure in thermal performance will likely require broader within-species sampling, multivariate environmental descriptors, and experimental designs that better approximate the combined factors experienced in the field. Given the growing interest in *Wolffia* as a potential human food source [4,13], extending similar temperature-screening efforts to this genus will be an important next step to broaden the applicability of our findings across duckweeds of high relevance.

## 4. Materials and Methods

### 4.1. Plant Material

The plant material consisted of 97 duckweed clones, distributed across three genera (*Spirodela*, *Landoltia* and *Lemna*) and 13 species (*S. polyrhiza*, *S. intermedia*, *La. punctata*, *L. disperma*, *L. gibba*, *L. minor*, *L. turionifera*, *L. minuta*, *L. valdiviana*, *L. obscura*, *L. aequinoctialis*, *L. perpusilla* and *L. tenera*), including 19 interspecific hybrid clones: 14 *L.* × *japonica* and five *L.* × *mediterranea* (Table S1). The clones used in this study were genetically characterized using the tubulin-based polymorphism (TBP) method, as previously described [37], and were obtained either from Professor Klaus J. Appenroth or from the IBBA-CNR-Milan duckweed collection (formerly the Landolt collection; GBIF dataset: https://doi.org/10.15468/hstsnr [63]. Duckweed clones were maintained aseptically on Schenk and Hildebrandt (SH) agar medium supplemented with 0.2% (*w*/*v*) sucrose (SH0.2), at 25 °C under a 16/8 h light/dark photoperiod and photon flux density of 31–34 μmol photons m^−2^ s^−1^.

### 4.2. Duckweed Temperature Screening Protocol

Duckweed plants were pre-conditioned to the liquid SH0.2 medium for 3 weeks under axenic conditions at 25 °C in a Sterivent high container 107 × 94 × 96 mm (Duchefa Biochemie, Haarlem, The Netherlands).

Growth performance was initially assessed under two temperature treatments: warm conditions (WC; 30/25 °C day/night) and relatively cool conditions (CC; 20/16 °C day/night). Temperature regimes were selected based on reported near-optimal/optimal ranges for duckweed growth; however, they cannot fully capture the thermal complexity of the clones’ sites of origin and were not intended to mimic the full variability of individual collection sites. In both treatments, plants were grown under a 16/8 h light/dark photoperiod and photon flux density of 31–34 μmol photons m^−2^ s^−1^.

For each condition, duckweed clones were tested in three or four independent biological replicates in a temperature-controlled growth chamber. Single healthy colonies for a total of eight fronds were randomly selected from pre-culture and transferred into a Sterivent high container containing 100 mL of SH0.2 growth medium. This initial density was chosen, after pilot experiments, to prevent very dense coverage and excess overlaps of fronds during the experiments, which could affect plant growth. Frond number refers to mother fronds and all recognizable daughter fronds.

After 14 days, plant material was harvested, blotted dry with paper towels, and lyophilized. Dry weight was then determined, and growth performance was expressed as relative growth rate (RGR, day^−1^), calculated as:

RGR = (lnDWt14 − lnDWt0)/t14 − t0

where DWt14 refers to the dry weight after 14 days of growth and DWt0 refers to the dry weight of the initial eight fronds [64]. To determine the weight of the initial 8 fronds, 40 fronds per each clone analyzed were counted in four replicates, lyophilized, weighed, and the dry weight was divided by 5.

Subsequently, 36 clones were selected to assess growth under extreme warm conditions (EWC; 35/30 °C, day/night), and 20 clones were selected to assess growth under extreme cold conditions (ECC; 16/12 °C, day/night). In both treatments, plants were grown under a 16/8 h day/night photoperiod and photon flux density of 31–34 μmol photons m^−2^ s^−1^.

### 4.3. Climate Data

We retrieved the world geolocalized 30-year (1970–2000) average, minimum, and maximum temperature records from the WorldClim 2.1 database [38]. The selected spatial resolution was 2.5 arc-minutes. The climate data of the clone sampling localities were obtained using the “point sampling tool” of Qgis 3.28 software.

### 4.4. Statistical Analysis

Data were analyzed using SPSS software (version 17, IBM, New York, NY, USA) and presented as mean ± standard error (±SE). A two-way ANOVA was used considering genotypes (grouped both at species and clone level), treatment (normal or extreme conditions) and bloc as independent variables. Genotype and treatment were considered as fixed factors, while bloc was considered as a random factor. When statistically significant interaction between independent variables was detected, simple main effects were calculated and reported. However, if interaction effect was not statistically significant, statistically significant main effects were reported.

## Data Availability

The original contributions presented in this study are included in the article.

## Supplementary Materials

The following supporting information can be downloaded at: https://www.mdpi.com/article/10.3390/plants15111649/s1, Figure S1: Density distributions of RGR values grouped by genus under warm (WC) and cool (CC) conditions. Dashed vertical lines indicate the mean RGR for each genus within each treatment; Figure S2: Mean RGR of clones under WC (A) and CC (B) plotted against the average temperature of clone origin. Blue circles indicate individual clones, the orange line represents the linear fit, and the red dashed line indicates the overall mean RGR. The shaded yellow area marks the selected temperature range. Pearson’s correlation coefficient is shown; Table S1: List of clones used in this study; Table S2: RGR of duckweed clones growth under WC and CC; Table S3: RGR of duckweed clones growth under EWC and ECC; Table S4: Pearson’s correlation coefficients (r) between mean RGR and the minimum, maximum, and average temperatures of the sites of origin for each species under WC and CC.

## Abbreviations

The following abbreviations are used in this manuscript:

RGR	Relative growth rate
WC	Warm condition
CC	Cool condition
EWC	Extreme warm condition
ECC	Extreme cool condition
