# Supplementary figures and images for: Temperature-Dependent Clonal and Species-Level Growth Variation in Spirodela, Landoltia, Lemna, and Interspecific Lemna Hybrids
Source: Plants (Basel). 2026 May 27;15(11):1649. doi: 10.3390/plants15111649 (PMC13259163; doi:10.3390/plants15111649)

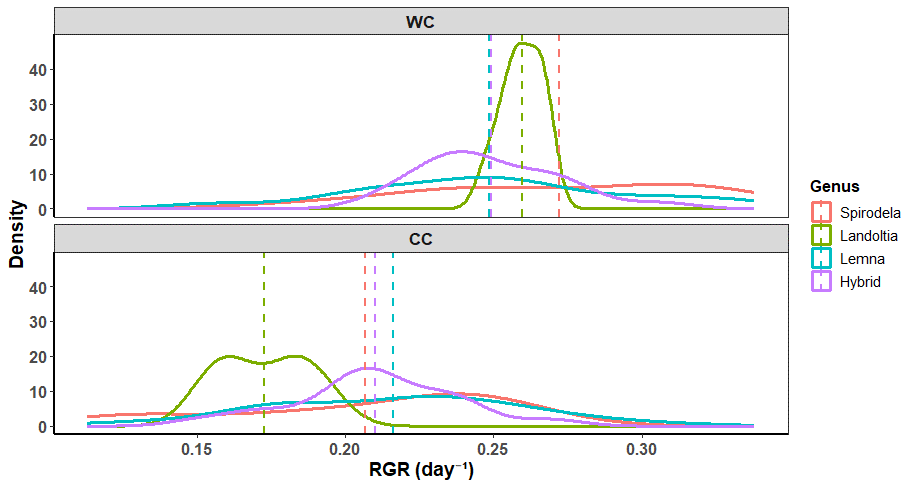

Supplement: Supplementary file 1 [file plants-15-01649-s001.zip › Supplemental Figures and Tables/Figure S1.png]

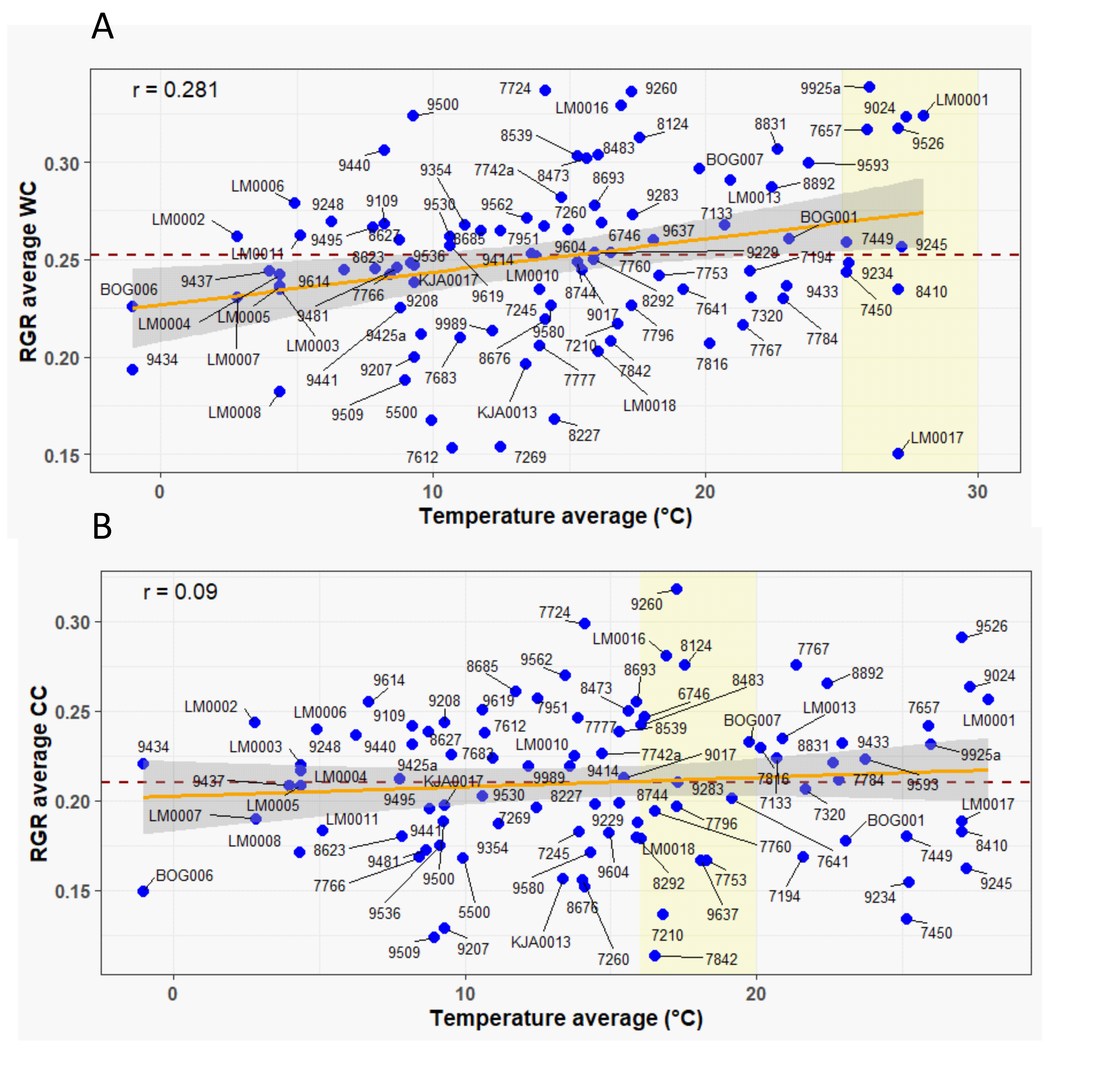

Supplement: Supplementary file 1 [file plants-15-01649-s001.zip › Supplemental Figures and Tables/Figure S2.PNG]
